# Extracts from a Letter Received from Dr. Marshall by a Friend in London

**Published:** 1801-04

**Authors:** J. H. Marshall

**Affiliations:** Valetta


### *Extracts from a Letter received from* Dr. MARSHALL *by a Friend in London.*

DEAR FRIEND,

SINCE our laft we have been exceedingly occupied in attending to the object of our miffion, and our prefence here has been exceedingly à propos, the Small-pox having been at Minorca, on board the fleet, and at Malta: The day after our arrival at this place, we inoculated feveral children in the

Foundling Hofpital, and from them others. In order to obtain the perfect confidence of the inhabitants, we determined to fubject fome of thefe children to the teft of the Small-pox; this was done in the prefence of the Governor, the Tuniffian Ambaffador, the Britifh Conful, many of the clergy, and the medical men of the ifland. Our fuccefs in this teft, as you may fuppofe, was complete; and now the Governor, &c. give to it the moft perfect confidence, with regard to its efficacy in ftopping the ravages of the Small-pox.

The Governor has had Dr. Jenner's work tranflated, and printed for the ufe of the inhabitants; and he has alfo eftablifhed an hofpital for the inoculation of the poor, under the title of the Jennerian Institution: Thus the true name is at laft given to this difcovery, which it ought at firft to have received, and which I hope now it will never lofe.

The Governor of the ifland, General Ball, is the idol of the Maltefe; he is eftablifhing a botanic garden, interefts himfelf in their hofpitals and public regulations, and among other conciliating meafures, boafts of the fervice England renders them by the introduction of the Cow-pox, and delights them by the offer of having the whole ifland inoculated. As the Small-pox is now amongft them, they have preffed in crouds, both the nobility and the mafs of the people. At the commencement of our inoculation, in going to the hofpital we formed a fmall proceffion; the Governor in his laced hat, the clergy in their canonicals, your friends Marfhall and Walker, and the medical men bringing up the train. The Governor has given us for our refidence the moft beautiful palace (heretofore the Grand Mafter's) of Valetta to refide in. The feafon here is moft delightful; it is now like May with you; green peas are juft coming in, cauliflowers, &c. are in perfection.

Amongft the fleet and army alfo our endeavours have been very fuccefsful. The Small-pox was ravaging, and had been very fatal; but it now feems to be extinct. Lord Keith and Sir R. Abercrombie have fhewed us every kindnefs; his Lordfhip particularly recommended the Jennerian Inoculation to the fleet, as you will fee by an extract from the Admiral's memorandum, in confequence of the Small-pox raging in the Alexander and other veffels.

*Foudroyant, Malta, Dec.* 9, 1800.

"The commander in chief thinks it neceffary to recommend to the refpective captains, an immediate application to Dr. Marfhall and Dr. Walker, whofe fafe and excellent mode of treatment has been experienced on board the Foudroyant and other fhips, in preventing the dreadful effects fo often attending the Small-

Small-pox, which may now ſo eaſily be avoided without danger or inconvenience.

"By command of the Vice Admiral,
"W. YOUNG."

It being impoſſible, on account of the ſhort ſtay of the fleet here, to complete the inoculation, it was neceſſary that one of us ſhould accompany it; and the enterpriſing ſpirit of Doctor Walker could not reſt till it was determined that he ſhould go. Since he went I have inoculated the whole of the troops left here under the command of General Pigot. I have had to combat the prejudices of the people, to convince them of its efficacy; and can ſay, that I have at laſt conquered. By a frigate which came in here laſt night, I learn that the fleet and the army were on the 13th inſtant in the Bay of Marmoriſſe, in Aſiatic Turkey; and that Dr. W. is very buſy in attending to his patients. The Dey of Algiers is very anxious to have our inoculation introduced amongſt them; ſo that there ſcarcely appears to be a proſpect of the termination of our labours. We have been made happy by meeting here ſeveral of our acquaintance on the medical ſtaff; and beg you to preſent our beſt reſpects to our friends in London. I am, &c.

*Valetta, Jan.* 21, 1801. J. H. MARSHALL.

